# The Effectiveness of Web-Based vs. Non-Web-Based Interventions: A Meta-Analysis of Behavioral Change Outcomes

**DOI:** 10.2196/jmir.6.4.e40

**Published:** 2004-11-10

**Authors:** Dean J Wantland, Carmen J Portillo, William L Holzemer, Rob Slaughter, Eva M McGhee

**Affiliations:** ^3^UCSF Comprehensive Cancer CenterSchool of MedicineUniversity of CaliforniaSan Francisco CAUSA; ^2^School of NursingUniversity of CaliforniaSan Francisco CAUSA; ^1^Department of Community Health SystemsSchool of NursingUniversity of CaliforniaSan Francisco CAUSA

**Keywords:** Web-based intervention, non-Web-based intervention, Web-based therapy, Internet, meta-analysis, patient outcomes, adults

## Abstract

**Background:**

A primary focus of self-care interventions for chronic illness is the encouragement of an individual's behavior change necessitating knowledge sharing, education, and understanding of the condition. The use of the Internet to deliver Web-based interventions to patients is increasing rapidly. In a 7-year period (1996 to 2003), there was a 12-fold increase in MEDLINE citations for “Web-based therapies.” The use and effectiveness of Web-based interventions to encourage an individual's change in behavior compared to non-Web-based interventions have not been substantially reviewed.

**Objective:**

This meta-analysis was undertaken to provide further information on patient/client knowledge and behavioral change outcomes after Web-based interventions as compared to outcomes seen after implementation of non-Web-based interventions.

**Methods:**

The MEDLINE, CINAHL, Cochrane Library, EMBASE, ERIC, and PSYCHInfo databases were searched for relevant citations between the years 1996 and 2003. Identified articles were retrieved, reviewed, and assessed according to established criteria for quality and inclusion/exclusion in the study. Twenty-two articles were deemed appropriate for the study and selected for analysis. Effect sizes were calculated to ascertain a standardized difference between the intervention (Web-based) and control (non-Web-based) groups by applying the appropriate meta-analytic technique. Homogeneity analysis, forest plot review, and sensitivity analyses were performed to ascertain the comparability of the studies.

**Results:**

Aggregation of participant data revealed a total of 11,754 participants (5,841 women and 5,729 men). The average age of participants was 41.5 years. In those studies reporting attrition rates, the average drop out rate was 21% for both the intervention and control groups. For the five Web-based studies that reported usage statistics, time spent/session/person ranged from 4.5 to 45 minutes. Session logons/person/week ranged from 2.6 logons/person over 32 weeks to 1008 logons/person over 36 weeks. The intervention designs included one-time Web-participant health outcome studies compared to non-Web participant health outcomes, self-paced interventions, and longitudinal, repeated measure intervention studies. Longitudinal studies ranged from 3 weeks to 78 weeks in duration. The effect sizes for the studied outcomes ranged from -.01 to .75. Broad variability in the focus of the studied outcomes precluded the calculation of an overall effect size for the compared outcome variables in the Web-based compared to the non-Web-based interventions. Homogeneity statistic estimation also revealed widely differing study parameters (Q_w16_ = 49.993, *P* ≤ .001). There was no significant difference between study length and effect size. Sixteen of the 17 studied effect outcomes revealed improved knowledge and/or improved behavioral outcomes for participants using the Web-based interventions. Five studies provided group information to compare the validity of Web-based vs. non-Web-based instruments using one-time cross-sectional studies. These studies revealed effect sizes ranging from -.25 to +.29. Homogeneity statistic estimation again revealed widely differing study parameters (Q_w4_ = 18.238, *P* ≤ .001).

**Conclusions:**

The effect size comparisons in the use of Web-based interventions compared to non-Web-based interventions showed an improvement in outcomes for individuals using Web-based interventions to achieve the specified knowledge and/or behavior change for the studied outcome variables. These outcomes included increased exercise time, increased knowledge of nutritional status, increased knowledge of asthma treatment, increased participation in healthcare, slower health decline, improved body shape perception, and 18-month weight loss maintenance.

## Introduction

A primary focus of self-care and self-management interventions is the encouragement of an individual's behavior change in the presence of a chronic illness or condition necessitating knowledge sharing, education, and understanding of the condition. There has been limited research comparing the use and effectiveness of Web-based interventions to non-Web-based interventions such as traditional face-to-face interactions and paper and pencil assessments. The introduction of the Internet into clinical practice as an information-sharing medium has brought about many opportunities for innovative interventions for individuals with chronic illnesses and their care providers. These interventions are often designed to address deficiencies in patient knowledge and chronic illness self-management skills. Improvements in these areas have been shown to lead to improved health outcomes. However, the extent of the benefits gained through the implementation of Web-based self-regulatory and behavior change interventions compared to non-Web-based interventions has not been fully ascertained. This meta-analysis was undertaken to establish any potential effect size differences between Web-based and non-Web-based interventions on selected patient behavior change outcomes.

In recent years, there has been an increase in the use of the Internet to gather, transform, and disseminate information that, in earlier years, was primarily done through the use of paper, in the form of books, pamphlets, instruction materials and so on. Internet users are seeking health information and healthcare services; 80%, or about 93 million Americans have searched for at least one of 16 major health topics online [1]. The Robert Wood Johnson Foundation (RWJF) has noted the increased use of Internet-based devices, cellular phones, and personal digital assistants (PDAs) creating opportunities for both patients and providers to benefit from access to e-Health applications. The RWJF has supported this trend by providing funding to study health behavior modification and chronic disease management in nontraditional settings through the use of e-Health technologies [2]. The use of computers to directly collect health assessment data from patients is a well-established technology that has been shown to produce reliable responses when administered over the World Wide Web [3]. In some circumstances, computer surveys have been shown to have advantages over face-to-face interviews. In one study, computer-based screening elicited more HIV-related factors in the health histories of blood donors than did standard questionnaire and interviewing methods [4]. Participant disclosure of high-risk sexual encounters has also been improved given the semblance of the more anonymous, Web-based data collection methodologies [5].

Computerized health behavior interventions are beneficial to patients/clients and healthcare providers. This is evidenced by structured reviews on the effectiveness devices such as kiosk-based computer assisted self-interviewing, interactive video, Internet applications, computer aided instruction, and the like in a variety of patient care settings. Balas and colleagues found that interactive patient instruction, education, and therapeutic programs helped individuals improve their health; at the same time, healthcare delivery processes were also improved [6]. Research studies suggest that education and knowledge sharing benefits can be achieved through computer-based education methodologies [6,7].

**Figure 1:**
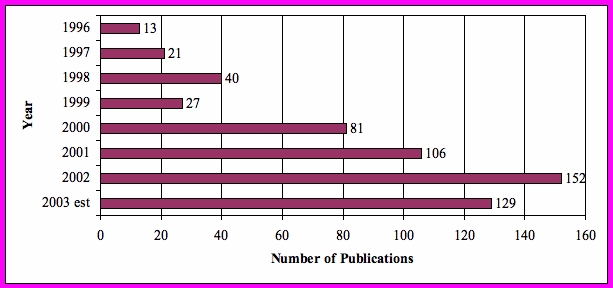
Search terms “Web-based Therapy” trended by year of publication

Interest in use of the Internet and Web-based interventions is increasing rapidly. In the 7-year period from 1996 to 2003, a total of 569 citations demonstrated a twelve-fold increase in MEDLINE publication citations for “Web-based therapies,” from 13 citations in 1996 to 152 citations in 2002. There has also been a steady increase in the number of citations in MEDLINE for the term “Web-based intervention,” further indicating interest in this research area for Web-based treatments. In addition to completed patient-focused, Web-based intervention studies, a large number of the publications are simply proposed or newly implemented studies. Many studies are based on therapeutic interventions that are provider focused and part of an implemented system incorporating the use of computerized medical records. Others include telehealth technologies that include highly technically interfaced lab values recorded within a case managed setting. Others discuss the variety and integrity of health-related Web sites (Figure 1).

## Methods

### Data Sources/Systematic Review

For identification of the relevant literature, a specific search strategy was performed using explicit inclusion criteria to avoid selection bias. A MEDLINE, CINAHL, EMBASE, ERIC, and PSYCHInfo search between the years 1996 and 2003 was conducted using keyword search terms of “computerized intervention,” “Internet intervention,” “Web-based therapy,” and “Web-based intervention.” The Cochrane Library collection was also accessed using keyword searches for “Web-based intervention” and “Internet intervention.” Searches in additional databases were done but revealed no new comparative Web-based published articles. A manual review of the reference lists of these articles was done to identify additional articles for possible inclusion. When an article was identified, it was compared against established inclusion/exclusion criteria to determine its suitability for the meta-analysis. The inclusion/exclusion criteria are presented in Table 1.

**Table 1:** Inclusion and exclusion criteria for the meta-analysis

Inclusion Criteria:
Publication date: January 1996 to December 2003. Comparison of a Web-based behavior or educational intervention, intended to influence behavioral change and/or self-efficacy health outcomes of participants compared to a non-Web-based method. Either randomized and controlled clinical trials or convenience samples Descriptive studies using a baseline and post study score(s) Clinic and clinic/home based studies Score of 12 or more on the Quality Rating Scale for the study (see Table 2).
Exclusion Criteria:
Publication date: prior to January 1996 Excluded studies: Non-Web-based Computer Assisted Instruction (CAI) studies Procedural methods citations (methods papers, non-implemented studies) Prospective non-implemented studies/citations Provider focused studies, no client participation Web site access only studies Professional practice studies Telephone based interventions Remote monitoring studies Interventions incorporating synchronous video communication Web-based intervention compared to another Web-based intervention Classroom or non-clinic/non-home location Score less than 12 on the Quality Rating Scale for the study (see Table 2).

### Quality Documentation of the Studies

The quality assessment of the included studies was based on the method used by Haynes and colleagues [8], with modifications to address the focus of this study on Web-based interventions. The compliance to standards for the studies is based on five criteria: (1) study design; (2) selection and specification of the study sample; (3) specification of the illness/condition; (4) reproducibility of the study; and (5) outcomes specification and the measurement instruments used/validity and reliability documentation of instruments. The sum of the variables result in a total score ranging from 0 to 18 (Table 2). Only studies with a quality documentation score of 12 or greater were retained for the meta-analysis.

**Table 2:** Quality evaluation of selected investigations (adapted from Haynes et al [8])

Study Characteristic	Evaluation Criteria	Scoring*
Study Design	1. Randomized trial 2. Non-randomized trial with control group 3. Descriptive/cohort study	3 points 2 points 1 point
Selection and specification of the study sample	1. Random selection with description of 4 to 5 demographic variables 2. Random sampling without sufficient description of the demographic variables 3. Convenience sampling with sufficient background information 4. Bonus point for a description of how many patients were excluded and reasons for exclusion.	3 points 2 points 1 point + 1 point
Specification of the illness/condition	1. Illness specified with reproducible inclusion/exclusion criteria. 2. Diagnostic criteria only were provided 3. Diagnosis only 4. Bonus point if all prior criteria were met and co-morbidities were described.	3 points 2 points 1 point +1 point
Reproducibility of the study	1. Description permits the reader to replicate the study 2. Results provided a standard for computing effect size (i.e., variable means, standard error, or standard deviation correctly stated).	1 point Yes: 3 points No: 0 points
Outcomes specification and measurement	1. Outcome measure is described and valid instrument use was clearly provided 2. Outcomes were not measured using valid and reliable instruments 3. Results did not match the described outcomes to be measured in the study	3 points 2 points -1 point
Maximum score		18 points

^*^ Only studies that scored 12 or higher were retained for meta-analysis

### Instrument Reliability and Validity

It is important to compare Web-based study instruments to their counterpart paper-based study instruments. Structured assessment instruments can be used to reliably measure a broad range of attributes of patient health and status. For comparative purposes in a meta-analysis, it is important to know the reliability of the measurement instruments with the reliability of the item measures reported in the publication. The validity and reliability of a Web-based measurement approach itself has not yet been adequately addressed. It cannot be assumed that the validity of an instrument based on its paper format and use in a specific research situation is transferable to the instrument's use in a Web-based format. Some instruments may be modified in ways that could change their meaning and accuracy, such that it might be inappropriate to compare data collected from different versions of the instruments (for example, provider administered assessments vs. self assessment). The ordering of the questions within an instrument can affect reliability and validity. In a Web-based format, the expected ordering may change and the ability to go back and review/change answers may need to be considered. The format of text can affect how the questions and instructions are interpreted. The use of bolding, italics, colors, fonts, and capitalization can affect the readability of items and change their phrasing. These can also draw attention to or from key parts of the instructions [9].

### Effect Size Calculation

A number of studies have been conducted having a measure that can be compared for its effect size in both a Web-based intervention vs. a non-Web-based intervention. Although the studies vary in the use of different outcomes that are used as measures for knowledge and/or behavior change, the construct of such change may be validly measured using meta-analytic techniques [10]. Although most studies had multiple outcomes from which to measure knowledge and/or behavior change, using several effect size calculations to represent results from each study outcome violates the rule of independence for statistical analysis, as these outcomes were obtained from the same sample of participants and were obtained in a similar setting. Multiple outcome effect sizes will also give disproportionate weight to studies with multiple groups and multiple scales compared to studies using fewer outcome measures.

Effect size was used to quantify the effectiveness of the Web-based intervention, relative to a non-Web-based comparison intervention. Effect size analysis was done to ascertain a standardized difference between the Web-based and non-Web-based groups, regardless of how the outcome was measured, by applying the appropriate meta-analytic technique. This analysis makes the assumption that individual studies are estimating different treatment effects and will observe the resulting effect size values and confidence intervals for distribution and variability. This check is done to evaluate if the effects found in the individual studies are similar enough that the combined effect size estimate is meaningful.

Hedges' d, a bias corrected modification of Cohen's d, was calculated to determine the magnitude of the difference between the mean of an intervention group and the mean of the control group, divided by a pooled standard deviation [10]. The calculations were based on the reported data in each of the studies that provided sample sizes, means, and standard deviations for each of the Web-based and non-Web-based intervention groups for the relevant effect (outcome) variables. A homogeneity statistic, Q_w_, was also calculated to determine whether the values of d used to calculate a mean effect size were consistent within the set of the reviewed studies. Heterogeneity is indicated when the Q_w_ statistic has a large, statistically significant value, suggesting that one or more features that were present in some studies and absent in others were affecting the magnitude of the effect sizes.

In controlled, repeated-measures studies, the effect size was calculated using the earliest time period for controls (non-Web-based intervention) and the final time period for controls then repeated for the intervention (Web-based intervention) groups, achieving one effect size for each group. The Web-based and non-Web-based group effect sizes were integrated to achieve one effect size for each study variable reviewed. In studies where standard deviations were not reported, but *P* values and/or z scores were provided, the Stouffer method for effect size calculation was used [11]. In studies having frequency or proportion data, the Mantel-Haenszel-Peto method was used to calculate the effect size between the Web-based and non-Web-based intervention groups [10]. For those studies that had multiple methodologies (i.e., multiple Web-based intervention groups compared to one paper-based group) or for those studies that used multiple paper-based methodologies (i.e., self-completion of a paper assessment and provider interview), the multiple group means were combined, the standard deviations were pooled, and effect size calculated. In those studies using a case/control, repeated measures design, the calculations for effect size and analysis of the effect sizes were performed using D-Stat Version 1.0 (Lawrence Earlbaum Associates, Inc., Hillsdale, NJ). Graphing was done using SPSS version 11.5 (SPSS Inc., Chicago, IL). Drop-line charts for individual groups using the variables for effect size and the low and high confidence interval values were graphed to provide visual representation effect sizes and associated confidence intervals.

Descriptive statistics were used to ascertain means and standard deviations as needed for aggregating the study data. Participant attrition rates in the longitudinal studies were calculated from the group N at the time of enrollment into the study until the time of the final reported follow-up period.

## Results

### Citation Searches

MEDLINE, CINAHL, EMBASE, PSYCHInfo, ERIC, and Cochrane Library, keyword searches resulted in 1518 citations. After reviewing for database redundancies in the citations, individual examination of the reference lists, and reviews of dissertations, a final review against the inclusion/exclusion criteria and quality documentation resulted in 20 studies selected for the instrument format analysis and the intervention-focused meta-analysis for behavior change outcomes. The selected studies were performed in the United States, France, Japan, Italy, Spain, Netherlands, Sweden, and Germany.

Exemplar studies, not selected for analysis, are summarized as follows: Studies that were Web-based to Web-based intervention comparisons [12-15]; 2) Studies that were descriptive of the functionality of a Web site [16,17]; 3) Studies that were provider focused [18]; 4) Pre/post intervention studies that only assessed the Web-based intervention [19-24]; 5) Studies that did not provide adequate information regarding either a change in outcomes or the comparative utility/validity/reliability of the Web-based tool [25-27]; and 6) Computer-assisted instruction (CAI) studies [28-30].

### Characteristics of the Reviewed Studies

Review of the selected articles revealed variation in design of the Web-based intervention studies. Because of the variation in the framework for these studies, two separate analyses were performed that: (1) evaluated studies that focused on a one-time, cross-sectional survey comparison of assessment instruments/methods when administered to Web-based and non-Web-based groups [3,31-34]; and (2) evaluated outcomes variables of intervention that best indicated knowledge and/or behavior change resulting from a Web-based intervention [35-51]. A summary of each study is shown in Table 3.

Aggregation of data from the 22 selected studies showed a total of 11,754 participants in both the Web-based and non-Web-based interventions at the time of inclusion into their respective studies. Of this total, 5,841 were women and 5,729 were men. The average age of participants was 41.5 years. For longitudinal studies, the average intervention duration was 27 weeks with a range from 3 weeks to 78 weeks. Attrition rates for the longitudinal studies revealed that both the intervention and control groups lost an average of 21% of the study participants over the duration of the studied interventions. (Table 4).

**Table 3:** Summary of reviewed studies**

**Author(s) and date**	**Intervention Focus**	**N and Study Characteristics**	**Conceptual Framework**	**Design**	**Variables/Behavior Change Variable**	**Study Findings**	**Reliability of Effect Variable Instrument**
Clarke G, Reid E, Eubanks D, O'Connor E, DeBar LL, Kelleher C, Lynch F Nunley S, 2002 [38]	Depression	N = 299 (I = 144, C = 155) 32-week study evaluating the effectiveness of a Web-based psycho educational tutorial intervention to reduce depression	Cognitive restructuring techniques	Longitudinal, Randomized study Repeated measures	IV = Intervention using tailored self- management or peer support therapy using cognitive therapeutic techniques DV = CES-D depression score change	No significant differences for the Internet program across the entire sample. Post-hoc, analyses revealed a modest effect among persons reporting low levels of depression at intake.	Center for Epidemological Studies-Depression (CES-D) 20-statement scale. Internal consistency from 0.85 to .90. Concurrent validity with Beck depression inventory, brief screen for depression.
Krishna S, Francisco BD, Balas A, Konig P, Graff GR, Madsen RW, 2003 [44]	Asthma Education	N = 228 (I = 121, C = 107) 52-week intervention comparing the use of IMPACT, an Internet enabled interactive asthma education program, to printed and verbal asthma education in a pediatric population, 18 years or younger.	Knowledge change leading to behavior change	Longitudinal, Randomized study Repeated measures	IV = Use of IMPACT, Web-based intervention DV = Childrens asthma knowledge, Caregivers asthma knowledge, days of asthma symptoms, medication use, ER /urgent care visits, missed school days, hospitalizations	Knowledge change was a primary indicator for program use and effectiveness. Multimedia education is a feasible adjunct that can be incorporated into a clinic visit. Increased asthma knowledge, decreased morbidity, and reduced use of ER services in IMPACT participants.	50-item asthma knowledge survey, 10 item asthma scenario survey. No validity or reliability documentation.
Celio AA, Winzelberg AJ, Wilfley D, Eppstein-Herald D, Springer EA, Dev P, Barr-Taylor C, 2000 [36]	Eating Disorders	N = 76 (Internet-student bodies (SB) = 27, class-based Body Traps (BT) = 25, wait-list/control (WLC) = 24) 8-week intervention and four-month follow up. Comparison of Web-based and classroom based psycho educational interventions to reduce body dissatisfaction and eating disorders/behaviors/attitudes.	Behavior change	Longitudinal, randomized study Repeated measures	IV = Web-based intervention, Class room intervention DV = Change in body satisfaction questionnaire scores, Eating disorder examination questionnaire, Eating Disorders Inventory (EDI)-Drive for thinness scale.	Internet intervention had a significant impact on reducing risk factors for eating disorders. No significant effects were found between the BT and WLC conditions	Body satisfaction questionnaire (BSQ) has internal consistency of .97, test-retest validity = 0.88, and concurrent validity coefficient = .66. At baseline measures, the EDE and BSQ showed spearman correlation = .86.
Harvey-Berino J, Pintauro S, Buzzell P, DiGiulio M, Casey-Gold B, Moldovan C, Ramirez E, 2002 [41]	Weight Control	N = 46 (Internet Support IS = 15, Traditional Support TS = 14, Control = 15) Web-based study, investigating the effectiveness of a weight maintenance program conducted over the Internet compared to in-person sessions. A 6-month clinical behavioral weight loss trial with in-person behavioral obesity treatment followed by a 12-month maintenance program conducted both in-person (frequent in-person support; F-IPS, minimal in-person support; M-IPS) and over the Internet.	Not discussed	Longitudinal, Randomized, 12 month maintenance program study	IV = use of Internet support method DV = body weight, dietary intake, energy expended in physical activity, attendance, self-monitoring, comfort with technology Behavior change exhibited by attendance in weight loss meetings	Attendance was lower in the Internet condition over the 12 months of maintenance than in the F-IPS condition. After 6 months, many in the IS want to meet face-to-face. The IS condition gained significantly more weight than the F-IPS group during the first six months of weight maintenance	No validity or reliability of assessment instruments was documented.
Oenema A, Brug J, Lechner L, 2001 [47]	Nutrition	N = 198, (I = 96, C = 102) Web-based tailored nutrition education program.	Weinsteins Precaution Adoption Process	Randomized trial Repeated measures (pre-post)	IV = Use of Web-based tailored nutrition education program DV = Validated food frequency questionnaire Behavior change exhibited by self report of awareness of personal dietary intake levels	Significant differences in awareness and intention to change were found between the intervention and control group at post-test. Tailored intervention was appreciated better, rated as more personally relevant, had more subjective impact on opinion and intentions to change than the general nutrition information.	Pearson correlations of about 0.7 for adults and 0.6 for adolescents were observed between fat scores derived from the Fat list and total and saturated fat intake in grams estimated by the 7-day diet records.
Harvey-Berino J, Pintauro SJ, Buzzell P, DiGiulio M, Gold BC, Moldovan C, Ramirez E, 2002 [42]	Weight Loss Maintenance	N = 122 (Internet = 40, Minimal in- person support = 41, Frequent in person support = 41) Sustained contact following a weight loss program	Not discussed	Longitudinal 18 month weight maintenance program	IV = Use of Internet support method DV = body weight, dietary intake, energy expended in physical activity, attendance, self-monitoring, comfort with technology Behavior change exhibited by 18 mos. weight loss maintenance.	Internet group reported increased peer support. Internet support not as effective as minimal or frequent intensive in-person therapist support for facilitating the long-term maintenance of weight loss.. Weight loss did not differ by condition during treatment The IS condition gained more weight than the F-IPS group during the first 6 months of weight maintenance and sustained lesser weight loss than control.	No validity or reliability of assessment instruments was documented.
Chou FY, 2003 [32]	HIV/AIDS	N = 359 (I = 122, C = 237) Self Care Symptom Management in individuals living with HIV/AIDS (SSC-HIVrev.)	Behavior Change	Convenience sample (Web version)	IV = Use of Wed-based version of symptom reporting tool DV = Help seeking strategies, personal network, information resources, Use of medications	Dissertation, participants in Web group reported decreased help seeking strategies, decreased spiritual strategies, and decreased personal networks compared to non-Web-based responders.	SSC-HIVrev. Part 1- 45 HIV-related symptoms cluster into 11 factor scores. Reliability .76 - .91; Part 2- 19 HIV-related symptoms that do not cluster into factor scores but may be of interest from a clinical perspective; Part 3- 8 items related to gyn symptoms for women. Cronbachs alpha = .94.
Marshall AL, Leslie ER, Bauman AE, Marcus BH, Owen N, 2003 [46]	Physical Activity Promotion	N = 655 (I = 327, C = 328) Eight week mediated physical activity Web-based intervention vs. eight week print based intervention	Trans-theoretical (stages of Change) Model	Longitudinal Randomized study	IV = Use of Web-based mediated physical activity (Active Living) intervention DV = Change in physical activity measured by the International Physical Activity Questionnaire (IPAQ) Short Past 7-day instrument.	Increase in total physical activity in the Print participants who were inactive at baseline. Decrease in average time spent sitting on a weekday in the Web group. No difference between Print and Web program effects on reported physical activity. The Print group showed slightly larger effects and a higher level of recognition of program materials.	No documentation of data supporting validity or reliability.
Gustafson DH, Hawkins RP, Boberg E, Pingree S, Serlin RE, Grazino F, Chan CL, 1999 [40]	HIV/AIDS	N = 204,( I =107 overall, C = 97) The Comprehensive Health Enhancement Support System (CHESS) developed for HIV/AIDS) Received system for 3 or 6 months; controls received no intervention of the CHESS system.	Behavior change	Longitudinal Randomized trial, Repeated measures Pre, intra, and post	IV = Use of CHESS system DV = QOL variables Medical outcomes study (MOS) short form Hospital resource utilization Behavior change exhibited by level of participation in healthcare	Intervention group had shorter ambulatory .care visits, more phone calls to providers, fewer & shorter hospitalizations compared to control during the computer implementation period. Post-implementation no difference in number and length of hospitalizations. Use of non emergency/ emergency were not significantly different between groups	Four subscales from the MOS 36, Physical function (α=0.87), cognitive function (α=.91), energy (α=0.85), depression (α=0.90)
Christensen H, Griffiths KM, Korten A, 2002 [37]	Cognitive Behavioral Therapy	Web-based sample of 1096 completed the Goldberg depression scale. Subanalysis also includes 49 students enrolled in an Abnormal Psychology course and local population survey of 2385 20-24 year olds Free access to MoodGYM Web intervention	Cognitive behavioral change	Descriptive Study	IV = Use of MoodGYM DV = Changes in depression and anxiety symptoms	20% of sessions lasted > 16 mins. Those who completed at least 1 assessment reported initial symptoms of depression and anxiety that exceeded those found in population-based surveys and those characterizing a sample of University students. Both anxiety and depression scores decreased significantly as individuals progressed through the modules	Goldberg Depression and anxiety Scales The full set of nine questions need to be administered only if there are positive answers to the first 4. When assessed against the full set of 60 questions contained in the psychiatric assessment they have a specificity of 91% and a sensitivity of 86%
Ritterband LM Cox DJ Kovatchev B McKnight L Walker LS Patel K Borowitz SM Sutphen J, 2003 [48]	Pediatric Encopresis	N = 24 (I = 12, C = 12) 3-week intervention for pediatric bowel training (Enhanced Toilet Training-ETT) to reduce defecation accidents called U-CAN-POOP-TOO. Evaluate the Internet version to overcome barriers of healthcare professional implementation of therapy alone.	Behavior change	Longitudinal study	IV = Use of Web-based U-CAN-POOP-TOO intervention for ETT DV = Reduction in number of defecation accidents, bathroom use change, encopresis knowledge questionnaire (EKQ), Virginia encopresis /constipation appreciation test (VECAT)	The Web participants showed improvement in reduced fecal soiling, increased toilet use, increased unprompted trips to the toilet. Both groups showed improvements in knowledge and toileting behaviors. Internet interventions may be an effective way of delivering sophisticated behavioral interventions to a large and dispersed population in a convenient format.	VECAT- consists of 18 pairs of drawings (9 pairs of bowel-specific and 9 parallel generic events), the child selects the picture in each pair that best describes him/herself. Authors state the VECAT has good internal consistency and testretest reliability.
Winzelberg AJ Eppstein D Eldredge KL Wilfley D Dasmahapatra R Dev P Barr-Taylor C, 2000 [51]	Eating Disorders	N = 60 (I = 31, C = 29) 8-week intervention and three-month follow up. Comparison of Web-based and classroom based psychoeducational interventions to reduce body dissatisfaction and eating disorders/behaviors/attitudes.	Behavior change	Longitudinal randomized study	IV = Web-based intervention, Class room intervention DV = Change in body satisfaction questionnaire scores, Eating disorder examination questionnaire, EDI-Drive for thinness scale	Evidence of feasibility for an Internet intervention to provide education via the Internet. At follow up, the intervention group showed improvement in body image and a decrease in the drive for thinness measures compared to controls.	Body satisfaction questionnaire (BSQ) has internal consistency of .97, test-retest validity =0.88, and concurrent validity coefficient = .66. EDI drive for thinness subscales have cronbachs alpha between .65 and .90.
Andersson G Stromgren T Strom L Lyttkens L, 2002 [35]	Tinnitus	N = 117 (I = 53, C = 64) Web-based cognitive behavioral therapy (CBT) to decrease distress caused by tinnitus.	Cognitive Behavioral Therapy	Longitudinal, randomized, Crossover design 6 month intervention, six month control	IV = Use of Web-based structured interview, treatment interactions, self-help program and weekly diaries DV = CBT Treatment efficacy evidenced by change in tinnitus reaction questionnaire, annoyance, anxiety sensitivity, depression scores	Reductions of tinnitus-related annoyance and anxious and depressive mood.	Tinnitus Reaction Questionnaire (TRQ) 26-item scale internal consistency of .96, test-retest correlation r=.88, Swedish version reported α = .97. Hospital anxiety and depression scales (HADS) show α=.82, α-.90 respectively.
Soetikno, RM. Mrad, R. Pao, V. Lenert, L., 1997 [33]	Ulcerative colitis (UC) and Quality of Life	N = 100 (I = 53, C = 47) Compared self-administered Internet based SF 36 and Irritable bowel QOL specific questionnaires (IBDQ) to paper-based administration.	Not discussed	Randomized Trial	IV = Use of Web-based assessment tool DV = Response demonstrating Validity of MOS 36 and IBD assessment surveys	Web-based scores on the IBPD tool were statistically different. Web participants had a wider range of scores and lower mean scores than clinic cases.	MOS-SF 36 Reliability cronbachs alpha: Phys. function .88-.93; Phys. role limits. 84-.96; Pain .80-.90, social function .68-.85; Mental health .82-.95; Emot. role limits 80-.96; Vitality .85-.96; Gen. health .78-.95.
Homer C, Susskind O, Alpert HR, Owusu M, Schneider L, Rappaport LA, Rubin DH, 2000 [43]	Asthma	N = 137, (I = 76,C = 61) children ages 3-12, 12-month study Effectiveness of interactive multimedia educational software program about asthma vs. control who reviewed printed educational materials with a research assistant.	Self efficacy theory	Longitudinal Randomized study	IV = Use of Interactive tool DV = Acute care use emergency department (ED), outpatient clinic (OP) clinic, reports of asthma severity. Parent/child knowledge of asthma.	No differences were demonstrated between the 2 groups in primary or secondary outcome measures. Both groups showed improvement in all outcomes. Increased knowledge after use of the computer program. Children reported having enjoyed using the program.	Child Health Questionnaire (CHQ-PF50) assessed functional status. 11 multi-item scales covering the physical, emotional and social well-being of children. Internal consistency alphas of .39-.96 (mean.72)
Lange A, Rietdijk D, Hudcovicova M, van de Ven JP, Schrieken B, Emmelkamp PM, 2003 [45]	Posttraumatic Stress Disorder	N = 184 (I = 122, C = 62) 5-week study consisting of two, 45 minute writing session per week consisting of self confrontation, cognitive reappraisal, and social sharing.	Behavior change	Longitudinal Randomized study	IV = Use of Web-based intervention DV = Change in Impact of Event (IES) scale, symptom checklist-90 scale	On most subscales, more than 50% of the treated participants showed reliable change and clinically significant improvement, The highest percentage change was found for depression and avoidance.	The IES (Dutch version by Kleber & Brom, 1986*). Uses a 5-point Likert scale on experiences for a given symptom during the past week. Cronbachs alpha .66 -.78 for the Avoidance subscale and .72 -.81 for the Intrusions subscale.
Strom L, Pettersson R, Andersson G, 2000 [50]	Recurrent Headache	N = 102 (I = 20, C = 25, dropout = 57) 6-week intervention of applied relaxation and problem solving to treat recurrent headaches while minimizing therapist contact.	Self-help	Longitudinal Randomized controlled study	IV = Use of the Web-based training program for headache relaxation techniques and headache problem solving DV = Headache index measure, # headaches, intensity, Becks Depression Inventory, Headache Disability Inventory	The Internet has the potential to serve as a complement in the treatment of recurrent headache. A significant reduction in the number of headaches for the treated participants.	No validity or reliability discussion.
Southard BH Southard DR Nuckolls J, 2003 [49]	2^0^ prevention heart disease	N = 106 (I = 53, C = 53) 6-month study comparing an Internet based program (SI) for nurse case managers to provide support, monitoring and education to patients with CVD. Tailored interactive home based system. Use was once a week for 30 minutes.	Not discussed	Longitudinal Randomized case control pre post study	IV = Use of Heartlinks DV = physiologic measure change, Minutes of exercise; MEDFICTS fat score; Depression score; Costs of care	Fewer CV events occurred in intervention (SI) than in control. Increased weight loss in SI group to control. Depression scores increased in both groups Minutes of exercise increased	Dartmouth (COOP) QOL assessment 8 factors and health status change score Becks Depression Inventory 21 items, Internal consistencies from .73 to .95.
Bell DS, Kahn CE Jr, 1996 [3]	Validity and Reliability assessment of Web-based MOS SF 36.	N = 4876 Web versions, 2471 MOS study Compared MOS SF 36 validity and reliability data of paper based documentation to Web-based version.	Not discussed	Convenience sample	IV = Use of Web-based SF 36 DV = Completion and Results of QOL subscales	97% of users completed the survey in < 10 minutes. Older participants required more time to complete the survey. Web participants had overall worse QOL subscale values	Subscale scores range from 0.76 to 0.90, similar to those of the MOS paper based reliability values.
Flatley-Brennan P, 1998 [39]	HIV/AIDS	N = 57 ( I = 37, C = 20) 25-week study demonstrating the use and effects of a specialized computer network among persons living with AIDS,	Rogers Diffusion of Innovation Theory	Longitudinal Randomized, Repeated measures study	IV = Home-based computer network use DV = Reduce social isolation improve confidence skills in decision-making no differential decline in health status among PLWA.	No significant difference between experimental and control groups Use of the system did reduce social isolation once participants levels of depression were controlled and that decision-making confidence improved as a function of number of accesses	Decision making confidence used a modified Saunders and Courtney 15 item - 22-item scale. (α=.80). Social isolation used Lins expressive social support scale (α=.88). Health status used 7 item Activities of Daily Living subscale (α=.76)
Wu AW, Yu-Isenberg K, McGrath M, Jacobson D, Gilchrist K, 2000 [34]	HIV/AIDS	N = 164 Touch-screen PC (n = 63,) Interview (n = 50), or self-administration (n = 51).	Not discussed	Randomized trial	IV = Use of touch screen in clinic kiosk PC to complete assessment tools DV = Reported measures from MOS-HIV, AIDS Clinical Trials Group (ACTG), Baseline Adherence and ACTG Symptom Distress	The reliability was noted to be comparable to face-to-face interview and self administration of the paper based tool.	Reliability of MOS_HIV α=0.69-0.94 for all subscales. Interclass correlations range between 0.54-0.88 for each subscale.
Bangsberg DR, Bronstone A, Hofmann R, 2002 [31]	HIV/AIDS	N = 110 Computer-assisted patient self report vs. provider estimate of HIV medication Adherence.	Not discussed	Convenience sample	IV = Use of Computer assisted, self-administered interviews (CASI) kiosk PC to complete survey tools. DV = Patient self report and provider medication adherence estimate, errors taking medication	54% of patients made at least one error in reporting their medication regimen. Providers tended to overestimate their patients' adherence and correctly classified only 24% of nonadherent patients at the 80% adherence level.	Validation of patient HIV medication self report done using the Aids Clinical trias Groups (ACTG) reasons for missing medications survey, viral load and CD4 lab values to assess detectable and non-detectable levels.

^**^ Intervention = I; Control = C; IV = Independent variable; DV = Dependent variable; PLWA = People living with AIDS;

^*^ Kleber RJ, Brom D. Traumatische ervaringen, gevolgen en verwerking (Traumatic events, consequences and processing). Lisse, The Netherlands: Swets & Zeitlinger; 1986

**Table 4:** Demographic characteristics of the cumulative studies

**Author**	Total N*	**Attrition % From Enrollment To Final Follow Up**				**Mean Age in years (Range)**	**Gender**	
							**Males**	**Females**
		**Intervention**		**Control**	**Study Duration**			
Andersson et al [35]	117	13%		7%	6 weeks	47.8	62	55
Bangsberg et al [31]	110	NA		NA	NA	46	96	14
Bell & Kahn [3]	4876	NA		NA	NA	38.2	2455	2421
Celio et al [36]	76	12%		31%	26 weeks	19.6 (18-36)	0	76
Christensen et al [37]	3530	48% reported combined			self paced	35.5	1567	1963
Chou [32]	359	NA	NA		NA	42.7	280	79
Clarke et al [38]	299	41% reported combined			32 weeks	43.7	73	226
Flatley-Brennan [39]	57	20%	12%		26 weeks	33.2	53	4
Gustafson et al [40]	204	12%	8%		26 weeks	34.6	184	20
Harvey-Berino et al [41]	46	4% reported combined			37 weeks	46.3 (31-60)	9	37
Harvey-Berino et al [42]	122	18% reported combined			78 weeks	48.4	18	104
Homer et al [43]	137	25%	20%		40 weeks	7.4 (3-12)	95	42
Krishna et al [44]	228	53%	58%		52 weeks	Not Specified	148	80
Lange et al [45]	184	53%	48%		5 weeks	47.8	Not Specified	
Marshall et al [46]	655	14%	19%		10 weeks	43	321	334
Oenema et al [47]	198	NA	NA		NA	44	75	123
Ritterband et al [48]	24	0%	0%		3 weeks	8.4	5	19
Soetikno et al [33]	100	NA	NA		NA	44.5 (midpoint) (35-54)	55	45
Southard et al [49]	106	4%	0%		52 weeks	62 (37-86)	80	26
Strom et al [50]	102	44% reported combined			6 weeks	36.7 (19-62)	33	69
Winzelberg et al [51]	60	23%	31%		20 weeks	20 (18-33)	0	60
Wu et al [34]	164	NA	NA		NA	41.5	120	44
Combined**	11754	21%	21%			41.5	5,729	5,841

^*^ Sample size (N) was derived from the number of cases newly enrolled into each study

^**^ Combined average age excluded: (1) Homer et al [43]; Ritterband et al [48]; Krishna et al [44]: subjects were all children 17 years of age or less. (2) Christensen et al [37], only those who participated in the completion of the Goldberg Depression Scale portion of the study. (3) Soetikno et al [33], only age range and midpoint were reported. Gender data were not reported by Lange et al [45]. Attrition rates were combined only for those specifying intervention/control.

NA=Non-longitudinal Study

### Knowledge and Behavioral Change Outcomes

Sixteen of the 17 studied effect outcomes revealed improved knowledge and/or improved behavioral outcomes for participants using the Web-based interventions. The individual effect sizes for each of the reviewed study variables for knowledge change and/or behavioral change showed effect sizes ranging from small (±.01 to .19) [36-38,41,44,46]; to moderate (±.20 to .47) [39,45,47,50,51]; to moderately large (.54 to .75) [40,42,43,49]. Of the 17 studied outcome variables, six showed that the positive effect sizes were statistically significant as seen by the confidence intervals being greater than zero [42-45,47,49] (Box 1). The one study favoring non-Web-based interventions did not show statistical significance [46]. There was no significant difference between the length of an intervention and effect size for the studied outcome.

Review of the forest plot graphical output figures showed a high degree of heterogeneity indicated by the confidence interval overlap (Box 1). Estimation of the homogeneity statistic was calculated and was statistically significant indicating variation between the 17 studies (Q_w16_ = 49.993, *P* ≤ .001). Sensitivity analysis to ascertain the studies with the greatest heterogeneity, revealed three standout studies [37,46,49].

**Effect size (ES) for outcome variables in the analyzed Web-based interventions compared to paper-based interventions (N = 17 Studies)**

**Table:** 

**Study #, Primary Author, Study Focus-Effect Variable**	**ES**	
		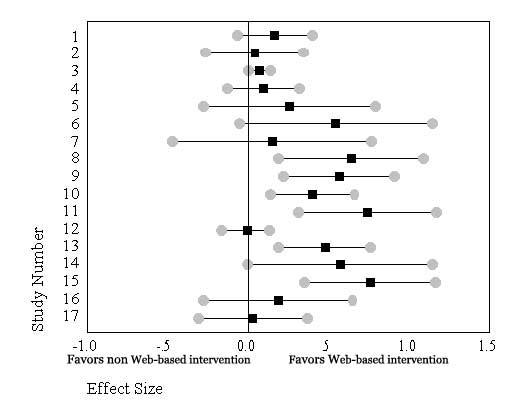
1. Andersson et al. Pre-post-follow up tinnitus reaction questionnaire [35]	.16	
2. Celio et al. Change in Body Shape Questionnaire [36]	.04	
3. Christensen et al. Goldberg Depression Scale-Mean Module 1 scores, gender combined [37]	.07	
4. Clarke et al. Depression (CES-D) score change [38]	.09	
5. Flatley-Brennan, HIV Use of ComputerLink networking -Slower health decline [39]	.25	
6. Gustafson et al. CHESS-HIV Change in participation in healthcare [40]	.54	
7. Harvey-Berino et al. Weight Loss Maintenance – pounds lost [41]	.15	
8. Harvey-Berino et al. 18-month weight loss maintenance [42]	.64	
9. Homer et al. Change in knowledge of asthma-treatment [43]	.57	
10. Krishna et al. Change in asthma knowledge scores in children [44]	.40	
11. Lange et al. Change in impact of event intrusion and avoidance combined score [45]	.75	
12. Marshall et al. Change in physical activity [46]	-.01	
13. Oenema et al. Tailored Nutrition Education – Intention to change diet [47]	.47	
14. Ritterband et al. Pediatric encopresis behavior change in bowel habit accidents [48]	.57	
15. Southard et al. Minutes of exercise [49]	.74	
16. Strom et al. Change in Headache Disability Inventory [50]	.19	
17. Winzelberg et al. Reducing risk factors for eating disorders - change in body shape questionnaire scores [51]	.03	

### Assessment Instrument/Methods Comparison

The five studies comparing assessment instruments/methods when administered to Web-based and non-Web-based groups revealed two studies showing moderate negative effect sizes (Wu -.24; and Soetikno -.22)[33,34] favoring the paper-based/traditional format. The remaining three instrument/method comparison studies showed small to moderate positive effect sizes ranging from .17 to .44. One of the five studies [31], showed a statistically significant effect size, indicated by zero being included in the confidence interval, the remaining four studies showed no statistically significant effect size comparison indicating little variability between the format of the instrument/method being either Web- or non-Web-based (Box 2). Analysis of homogeneity of these five studies revealed a statistically significant Q value (Q_w4_ = 18.238, *P* ≤ .001).

**Effect size (ES) evaluation of studies assessing instruments/methods when administered to Web-based and non-Web-based groups (N = 5 Studies)**

**Table:** 

**Study #, Primary Author, Study Focus-Effect Variable**	**ES**	
		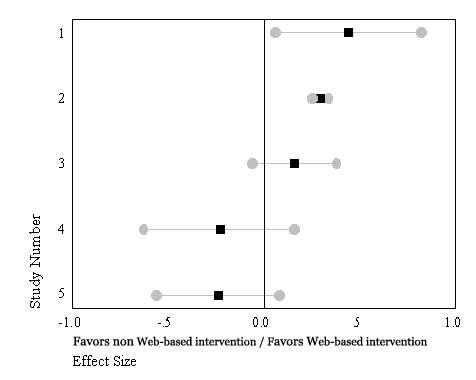
1. Bangsberg et al.– CASI Self Report HIV Medication Adherence [31]	.44	
2. Bell et al. SF 36 QOL All Subscales [3]	.29	
3. Chou. HIV symptom self care –Taking medications, prescription, OTC medications [32]	.16	
4. Soetikno et al. SF-36 QOL-All scales [33]	-.23	
5. Wu et al. MOS-HIV QOL Survey-All scales [34]	-.24	

## Discussion

### Advantages for the Use of Web-based Interventions

The management of any chronic disease should be personalized to an individual, as the person is ultimately responsible for the success of the intervention. Self-management of a chronic condition and contribution to disease management has demonstrated improved results and adherence to treatment regimens [52]. Consequently, Web-based interventions should be designed to allow individuals to tailor the intervention to their specific needs. With the advent of high-level Web programming languages, intended to provide effective data and information provision and retrieval, the flexibility to provide interactive and responsive programs for use on the Internet is increasing. This is conducive to the incorporation of interactive and continuous self-monitoring, feedback and information exchange that is certain to play an increasingly important role for this patient care need.

### Comparative Intervention Studies

Although the studies vary across many clinical areas of interest, there is a consistency of the selected outcome variables being targeted to require either or both an individual's knowledge and behavior change to achieve the outcome. The review of the individual study effect size comparisons in the use of Web-based compared to non-Web-based interventions showed an improvement in individuals using Web-based interventions to achieve behavior change for the studied outcome effect variables. The broad variability in the focus of the studied outcomes precluded the calculation of an overall effect size for the compared outcome variables in the Web-based when compared to the non-Web-based interventions. Additionally, a homogeneity statistic estimation also revealed widely differing study parameters (Q_w16_ = 49.993, *P* ≤ .001). Sensitivity analysis ascertained three studies with the greatest heterogeneity [37,46,49], these studies were not excluded from the analysis as their contribution to the research using Web-based and non-Web-based interventions showed significant findings. There was no significant difference between study length and effect size in the longitudinal studies.

### Assessment Instrument/Method Comparison Studies

A comparison of the five Web-based instruments and the non-Web-based instruments shows the variability between the formats of the instrument to be moderate to small. The effect size analysis confirms the respective authors' findings in each of their studies. For the studied instruments, the Web-based instruments produced valid and reliable results. These studies revealed effect sizes to range from -.25 to +.29, only one of which was statistically significant, favoring Web-based interventions. In the studies that measured the use of quality of life (QOL) instruments such as the MOS-HIV and the SF-36, it should be noted that in the Bell and Kahn study [3], there was no specification of any predisposing illness in the Web-based intervention group. In the non-Web-based population, the scores reported by the authors of the comparative study [53], were combined from studies with participants having varying illnesses, which may account for this comparison group having worse SF-36 scores than the anonymous comparison group. Further, these QOL instruments may not be sensitive enough to capture the illness severity of the subscales for Web-based clients. Floor effects have been reported for the SF-36 for those with severe illness related impairment [54]. Conversely, ceiling effects may be present if the Web-user is doing well and not experiencing levels of debilitation due to symptoms. The MOS-HIV and SF-36 may not possess sufficient sensitivity to change to adequately reflect the symptom experience and management of symptoms in ongoing tailored interventions requiring daily or weekly input.

### Demographic Characteristics

Most of the studies explained the possibility of demographic differences (i.e., culture, age, gender, ethnicity, and/or income) in their study intervention populations. Some studies controlled for the possibility of these differences [40], while others provided training to the Web-based intervention participants [34,43,47]. In the reviewed studies, the average age of the study participants was 41.2 years, which is relatively young. It is likely that this is not the same population who are living with many chronic illnesses. Most of the studies did not discuss issues such as ethnicity, income level, or homelessness, which are important when considering the use of a Web-based technology to deliver an outpatient intervention. All but one of the studies [45] did report gender, but overall, the differences between participation of men and women were not large in the studies. Two studies looked at HIV interventions and had a preponderance of men (N = 237) with an average age of 37.5 years [34,40]. The studies by Bell et al and Christensen et al [3,37] were open access Web sites and had lower average ages compared to their non-Web-based control groups.

### Dose of an Intervention

There are tools available that ascertain use of a Web site, visits to a various pages on the site, and paths to trace links and usage patterns by the user. These are useful to determine the dose of the Web-based intervention. Based on the individual's response, how much intervention that is needed by an individual can be tailored and varied. In the reviewed studies that discussed their Web site use statistics, (see Table 4) there was large variability in the average intervention time and the number of logons to the sites. The average session site time of 19.3 minutes should be considered in context of the attributes of the individual using the Web site and the burden the intervention may place on the individual to complete the items and contribute any necessary interactive responses. The burden to complete the needed information throughout the site may be relieved by increased interactivity to create and maintain interest in the site. Interactivity may help reduce attrition of Web users and provide benefits in producing positive behavioral change.

**Table 7:** Web site usage statistics

**Author**	**Focus/Intervention**	**Average Intervention Time/ site session (in minutes/person)**	**Web Site session logon average (/ person)/ study duration (weeks)**
Andersson et al [35]	Tinnitus	Not discussed	Not discussed
Bangsberg et al [31]	Computer Assisted Self-Reported Medication Adherence	Not discussed	Not discussed
Bell and Kahn [3]	Quality of life using the SF-36	4.5 min/p	Not discussed
Celio et al [36]	Eating Disorders	Not discussed	Not discussed
Christensen et al [37]	Depression and Anxiety Prevention in the General Public	9.47 min/p	280 person/6 wks
Chou [32]	HIV Self Care Symptom Management - Medication Taking	Not discussed	Not discussed
Clarke et al [38]	Depression	Not discussed	2.6 person/32 wks
Flatley-Brennan [39]	Use of ComputerLink Networking in Persons with HIV	12.5 min/p	188 person/26 wks
Gustafson et al [40]	CHESS - In Persons With HIV	Not discussed	1008 person/36 wks
Harvey-Berino et al [41]	Weight Loss Maintenance	Not discussed	Not discussed
Harvey-Berino et al [42]	Weight Loss Maintenance	Not discussed	Not discussed
Homer et al [43]	Asthma Education Program	Not discussed	Not discussed
Krishna et al [44]	Asthma Education Program use by children	Not discussed	Not discussed
Lange et al [45]	Post Traumatic Stress Disorder	45 min/p	10 person/5 wks
Marshall et al [46]	Physical Activity	Not discussed	Not discussed
Oenema et al [47]	Tailored Nutrition Education	Not discussed	Not discussed
Ritterband et al [48]	Encopresis	Nor discussed	14 person/3 wks
Soetikno et al [33]	Ulcerative Bowel Syndrome	Not discussed	Not discussed
Southard et al [49]	Prevention of Secondary Cardiovascular Disease	25 min/p	47 person/26 wks
Strom et al [50]	Headache Disability	Not discussed	Not discussed
Winzelberg et al [51]	Eating Disorders	Not discussed	Not discussed
Wu et al [34]	HIV Touch Screen MOS HIV Administration	Not discussed	Not discussed
Combined		19.3 min/p	

### Variation in Study Validity

The comparative intervention studies invited participation into their studies either by e-mail or by in-person enrollment [35,36,38,40-43,45-51]. In all these studies, personal information for continued contact (i.e., telephone number, mailing and e-mail addresses) was obtained. This is in contrast to some studies in the instrument comparison study group where self-identification and e-mail participation was obtained for the Web-based participation and the participants were anonymous [3,32,37].

Selection bias may be introduced, as it is possible that Web-savvy clients and researchers may have differing attributes from non-Web-familiar clients and researchers. Familiarity with the use of computers and the Internet may lead to self selection in the use of these technologies. Conversely, non-familiarity with computers and the Internet may lead others to refrain from participation, increasing attrition in these interventions. In addition, some of the anonymous Web-based participants who may have completed the assessments may not have truly met the criteria for the study. Additionally, publication bias is possible as there is the possibility of missed publications in spite of the systematic literature review process.

### Conclusion

There is substantial evidence that use of Web-based interventions improve behavioral change outcomes. These outcomes included increased exercise time, increased knowledge of nutritional status, increased knowledge of asthma treatment, increased participation in healthcare, slower health decline, improved body shape perception, and 18-month weight loss maintenance. Those interventions that directed the participant to relevant, individually tailored materials reported longer Web site session times per visit and more visits. Additionally, those sites that incorporated the use of a chat room demonstrated increased social support scores. The long-term effects on individual persistence with chosen therapies and cost-effectiveness of the use of Web-based therapies and hardware and software development require continued evaluation.
